# Supplementary material for: Illuminating Glucomannan Synthases To Explore Cell Wall Synthesis Bottlenecks
Source: ACS Synth Biol. 2025 Jul 8;14(7):2472–9. doi: 10.1021/acssynbio.5c00181 (PMC12281619; doi:10.1021/acssynbio.5c00181)
Supplement: Supplementary file 1 [file sb5c00181_si_001.pdf]

Supporting Information

Illuminating Glucomannan Synthases to Explore Cell Wall Synthesis Bottlenecks

Annika Grieß-Osowski[1,†]*, Madalen Robert*[1,2]*, Moni Qiande*[2]*, Stefanie Clauss*[1]*, Cătălin Voiniciuc*[1,2]*

[1] Independent Junior Research Group–Designer Glycans, Leibniz Institute of Plant Biochemistry, 06120 Halle (Saale), Germany

[2] Horticultural Sciences Department, University of Florida, Gainesville, FL 32611, USA

† Current Address: Heinrich Heine University, Biological Data Science, Universitätsstraße 1, 40225 Düsseldorf, Germany

Corresponding Author:

Cătălin Voiniciuc – Horticultural Sciences Department, University of Florida, Gainesville, FL 32611, USA; https://orcid.org/0000-0001-9105-014X; Phone: +1 352-273-4782; Email: cvoiniciuc@ufl.edu

Supporting Information

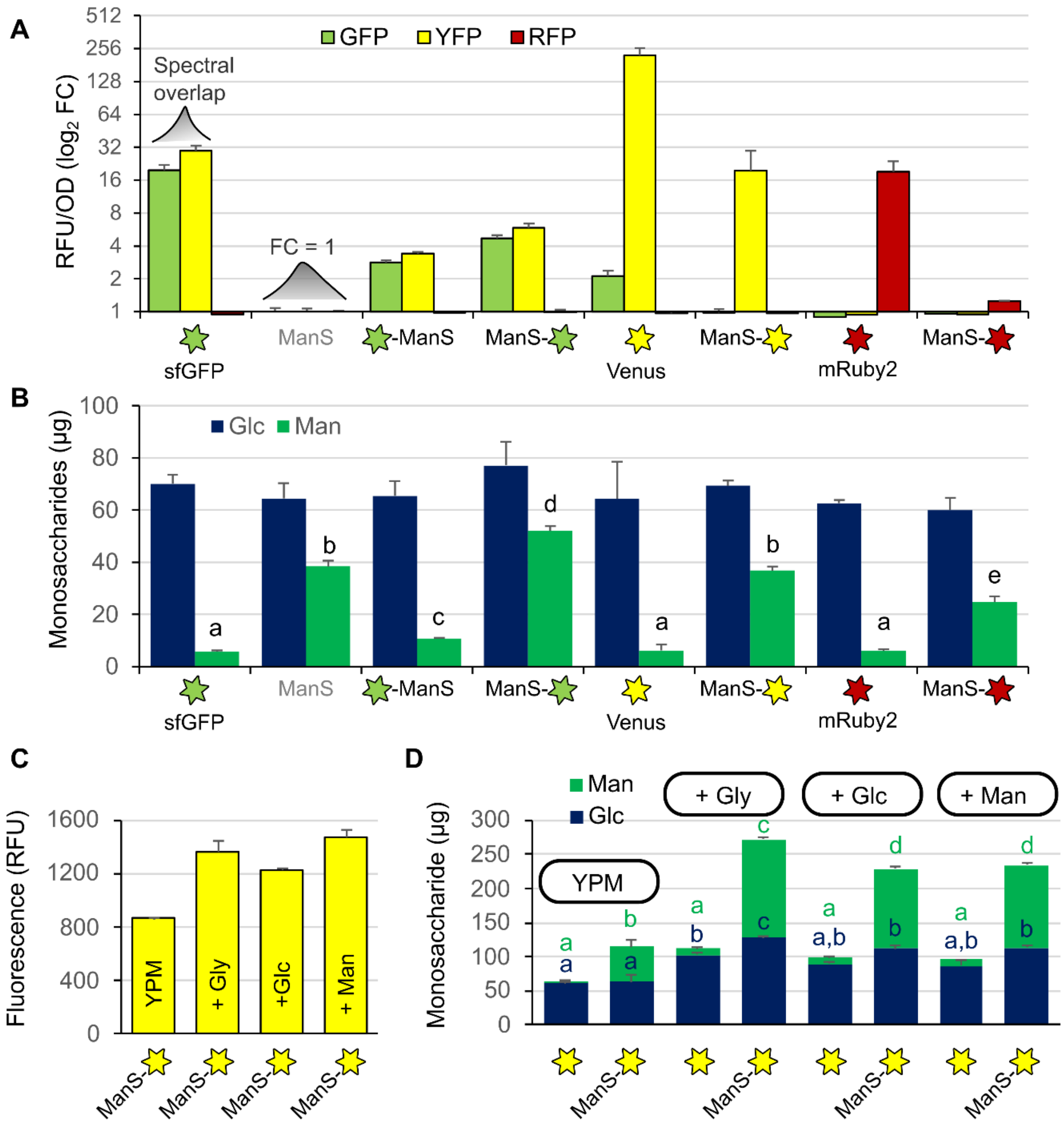

Figure S1. *Pichia* expressing ManS with or without C-terminal FPs. (A) Log2 fold changes (FC) in relative fluorescence units normalized to OD600 biomass. FC was set to 1 for the untagged ManS control in each of the three fluorescence channels that were analyzed with a plate reader. GFP and YFP channels show spectral overlap, particularly for the sfGFP tag. Yeast strains were cultivated for 24 h in BMGY medium for biomass production followed by 24 h growth in BMMY. (B) Composition of AKI wall polymers from samples in (A). (C) Relative fluorescence of ManS-Venus in YPM medium alone, or in YPM medium supplemented with 0.5% w/v Glycerol (Gly), Glucose (Glc) or Mannose (Man). (D) Composition of yeast AKI wall polymers from Venus and ManS-Venus lines after the feeding strategies shown in (C). For all panels, data show the mean + SD of at least 3 biological replicates and letters denote for significant changes (one-way ANOVA with Tukey test, $P < 0.05$).

Supporting Information

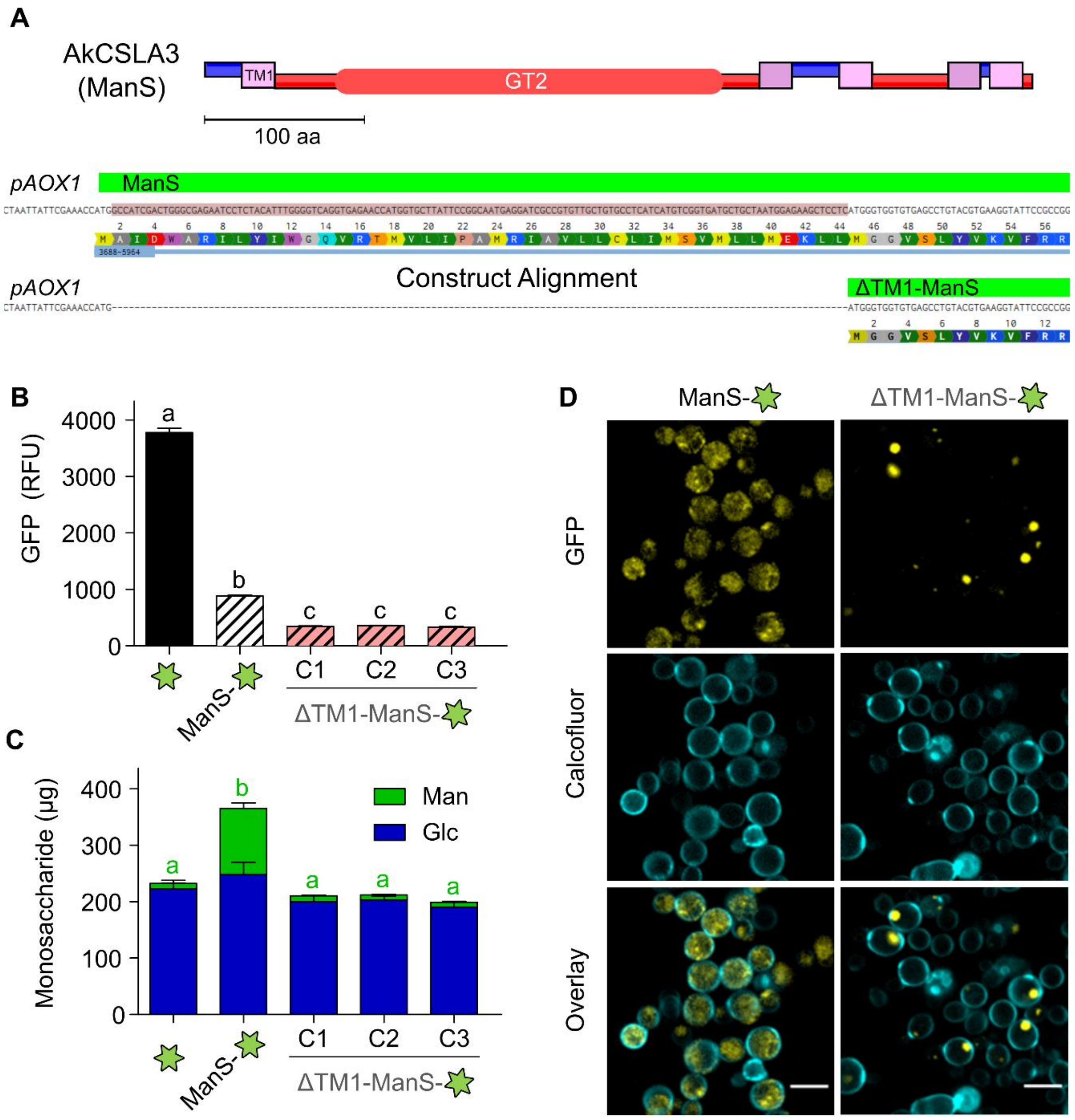

Figure S2. Impact of N-terminal region on ManS-sfGFP expression and activity. (A) Topology of AkCSLA3 (ManS in this study) adapted from an earlier publication.[1] Amino acids (aa) 24 to 44 are predicted to encode the first transmembrane domain (TM1). A plasmid sequence alignment from Benchling shows the *pAOX1* promoter followed by the ManS coding sequence. (B) Relative fluorescence units (GFP/OD) of sfGFP-tagged constructs using BB3_aZ integrative vectors and (C) composition of AKI wall polymers. *Pichia* cultures were cultivated in YPM + G for 48 h. Data show the mean + SD of 3 biological replicates and different letters denote significant changes (one-way ANOVA with Tukey test, $P < 0.05$). For the TM1 deletion constructs, three independent colonies (C1 to C3) were analyzed. (D) Localization of sfGFP-tagged ManS proteins with or without the N-terminal region. *Pichia* cell walls were counterstained with calcofluor. Scale bars: 5 µm.

Supporting Information

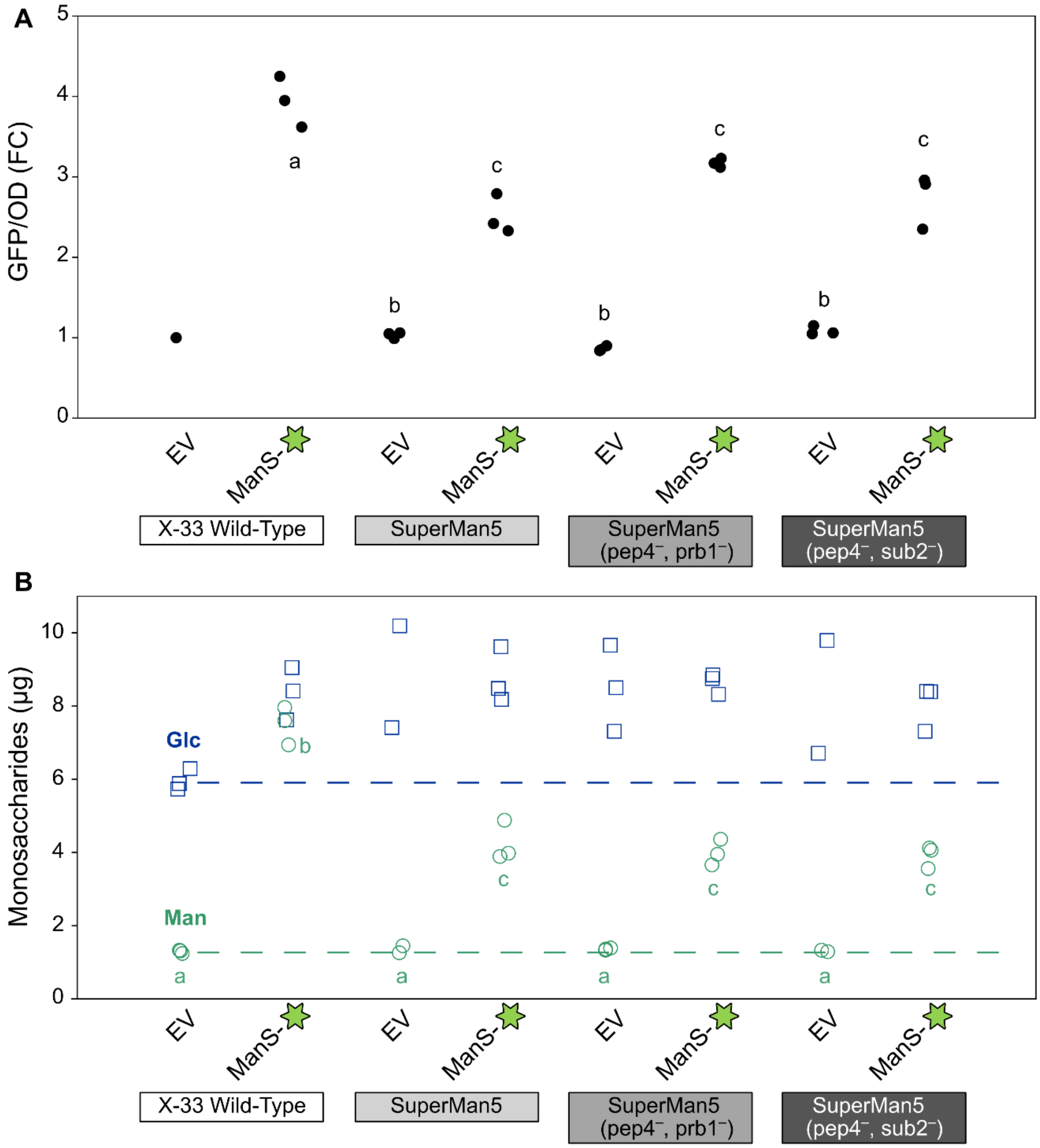

Figure S3. ManS-sfGFP performance in three SuperMan5 strains. (A) Fold changes (FC) in fluorescence (GFP/OD600) were normalized to pPICZ B empty vector (EV) control, which was set to have FC = 1 in the X-33 background after growth for 42 h in BMMY. The Superman5 (pep4⁻, prb1⁻) and Superman5 (pep4⁻, sub2⁻) strains are protease-deficient. (B) Monosaccharide composition of AKI polymers in equal aliquots. Graphs show independent colonies for each vector in four different *Pichia* backgrounds. Letters in (B) denote significant changes in Man levels (one-way ANOVA with Tukey test, $P < 0.01$).

Supporting Information

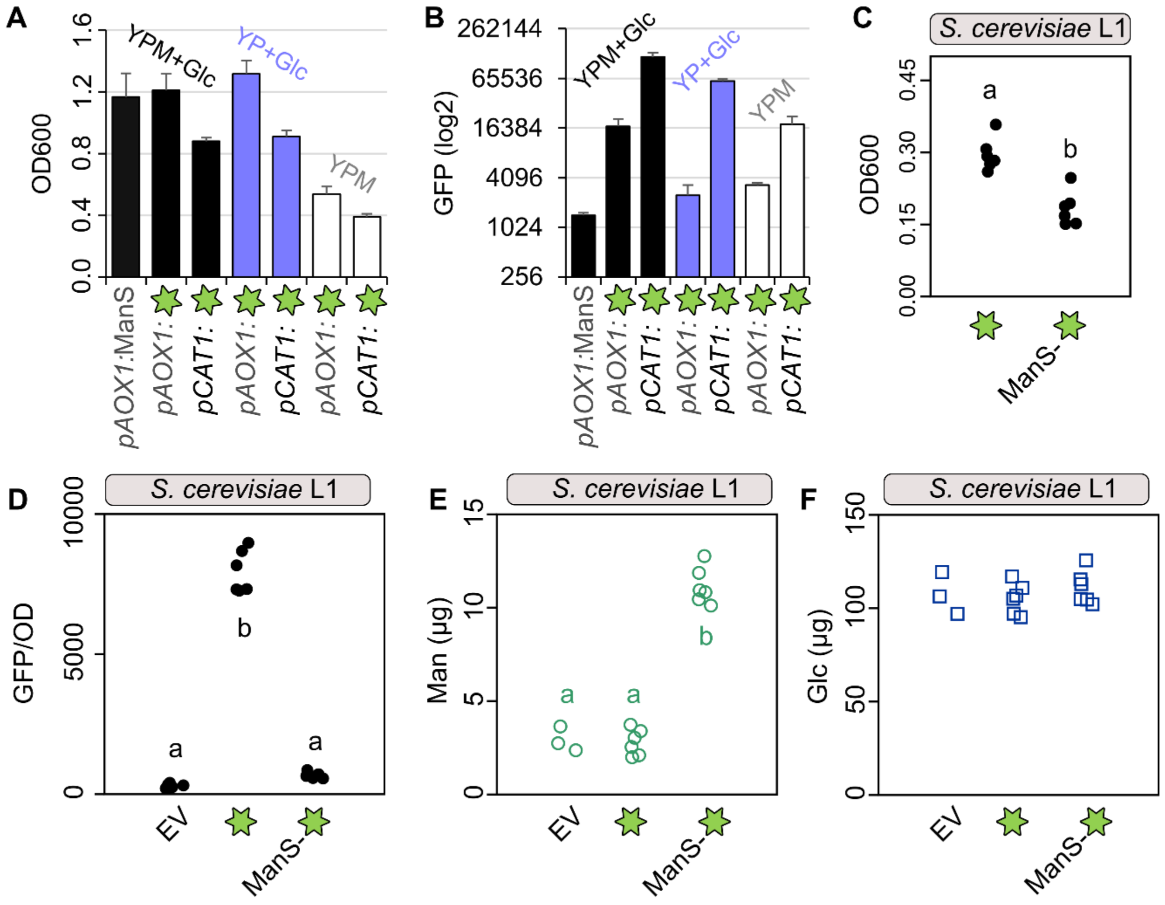

Figure S4. Feeding strategies and episomal vector performance in two yeast species. (A) Optical density and (B) GFP fluorescence (logarithmic scale) of *pAOX1* versus *pCAT1* episomal *Pichia* strains after 48 h of growth in YP-based media supplemented with 2% v/v methanol (M) and/or 0.5% w/v Glc. The *pAOX1:ManS* episomal construct is untagged and serves as a negative control for fluorescence. Bars show mean + SD of three biological replicates per genotype. (C) Optical density of *Saccharomyces cerevisiae* after 24 h of direct cultivation in DO^{Ura-} supplemented with 2% (w/v) galactose to induce protein expression of an L1 episomal vector. (D) Relative fluorescence units of *S. cerevisiae* L1 empty vector (EV), sfGFP and ManS-sfGFP after biomass accumulation in YPD for 48 h, followed by 24 h of induction in DO^{Ura-} with 2% (w/v) galactose. This two-step cultivation strategy (corresponding to Figure 5C) produces plant mannan (E) without compromising biomass accumulation (F), as shown by consistent levels of yeast glucans. In (C) to (F), dots represent independent colonies and letters mark significant differences obtained by one-way ANOVA with Tukey's pairwise ($P < 0.05$).

Supporting Information

Table S1. Yeast strains and vectors used in this study. For C-terminal fusions in the pPICZ B vector (EasySelect Pichia Expression kit, ThermoFisher Scientific), the fluorescent proteins (FPs) were inserted using Eco72I and XhoI sites and classical restriction enzyme (RE) cloning. The ManS coding sequence was trimmed to remove its stop codon and ensure in-frame FP fusion using Bsp119I and Eco72I RE cloning in pPICZ B + FP vectors. Sequences were domesticated and assembled into GoldenPiCS vectors, as directed by the original publication[2], using Golden Gate cloning. *Pichia* episomal vectors were assembled using *pPAP002* or a new *L1_2F_ePH* vector modified from *pPAP002* to accept any MoClo promoter-terminator combination. For *Saccharomyces* cerevisiae expression, genes were assembled in the L1 episomal vector *pAGT572_Nemo*,[3] containing a galactose-inducible promoter *pGAL1*.

**Yeast strains** and plasmids	**Reference**	**Assembly**	**Promoter**
Pichia pastoris X-33	EasySelect Pichia kit	-	-
***Pichia pastoris* SuperMan5 strains**	RCT's Pichia Glycoswitch[4]	-	-
pPICZ B	EasySelect Pichia kit	RE cloning	Inducible, pAOX1
pPICZ B + sfGFP	prior work[5]		
pPICZ B + sfGFP-ManS	prior work[5]		
pPICZ B + ManS-sfGFP	this study		
pPICZ B + Venus	this study		
pPICZ B + ManS-Venus	this study		
pPICZ X + mRuby2	this study		
pPICZ X + ManS-mRuby2	this study		
BB3aZ_14 + pAOX1:ManS-sfGFP:RPP1Btt	prior work[1]	GoldenPiCS	Inducible
BB3aZ_14 + pAOX1:ΔTM1-ManS:sfGFP-RPP1Btt	this study		Inducible
BB3rN_14 + pGAP:ScGOS1-mRuby2:ScCYC1tt	this study		Constitutive
pPAP002	prior work[3]	Plant MoClo	Inducible, pCAT1
pPAP002 + sfGFP	this study		
pPAP002 + ManS	this study		
L1_2F_ePH	this study		any promoter
L1_2F_ePH + pAOX1:sfGFP:RPP1Btt	this study		Inducible, pAOX1
L1_2F_ePH + pAOX1:ManS:RPP1Btt	this study		
Saccharomyces cerevisiae BY4742	prior work[6]	-	-
pAGT572_Nemo	prior work[3]	Plant MoClo	Inducible pGAL1
pAGT572_Nemo + sfGFP	this study		
pAGT572_Nemo + ManS-sfGFP	this study		

Supporting Information

Table S2. Primers used for cloning and genotyping. Restriction enzyme recognition sites are in bold and Golden Gate fusion sites are underlined. The insertion sites in GoldenPiCS vectors are flanked by commonly used M13 sequencing/genotyping primers. The pPICZ B + ManS-sfGFP was cloned by amplifying an unpublished pPICZ B + AkCSLA3-2A-sfGFP plasmid using 5’ Phosphorylated, HPLC-purified primers, and circularizing the resulting amplicon.

Target and Application	Forward Primer	Reverse Primer
pPICZ B + ManS-sfGFP	[5' Phos] TTTTCACTAGGAACAAAGGTGCCA	[5' Phos] atgcgtaaaggcgaagagctg
C-ter Venus for pPICZ	caa**CACGTG**tcatgtctaaaggtgaagaattattcactgg	gac**CTCGAGTC**Atttgtacaattcatccataccatg
ManS for C-ter fusions in pPICZ	a**TTCGAA**acgatatggccatcgactg	caa**CACGTG**tcatgtctaaaggtgaagaattattcactgg
C-ter mRuby2 for pPICZ	caa**CACGTG**tcatggtgtccaaaggagagga	gtg**CTCGAG**ctacttatacaattcatccataccaccg
Venus for BB1	cgct**GGTCTC**aCATGtctaaaggtgaagaattattc	acga**GGTCTC**gaagcttatttgtacaattcatccatacc
mRuby2 for BB1	atct**GGTCTC**aCATGgtgtccaaaggagaggag	acga**GGTCTC**gaagcttacttatacaattcatccataccaccg
Fs2-ScGOS1-new for BB1_23	acga**GGTCTC**aCATGAGCTCACAACCGTCTTTCG	gctc**GGTCTC**aCTCCCCATGTGAAAAACAAAAACAG
new-mRuby2-Fs3 for BB1_23	atat**GGTCTC**aGGAGTGTCCAAAGGAGAGGAGTTA	acga**GGTCTC**gaagcTTActtatacaattcatccataccaccg
Fs3-sfGFP-new	atat**GGTCTC**aGCTTcccgtaaaggcgaagagctg	taat**GGTCTC**actatttgtacagttcatccataccatgc
new-RPP1Btt-Fs4	atat**GGTCTC**aatagggatgatactttaattgatgc	tcga**GGTCTC**cAGCGgctcctctagataccatcaag
pAOX1 for L0_12	ttc**GAAGAC**tcGGAGagacgaaaggttgaatgaaac	cgc**GAAGAC**gccattgtttcgaataattagttgtttttg
RPP1Btt for L0_34	ttc**GAAGAC**tcGCTTgcttggatgatactttaattg	att**GAAGAC**tgagcggctcctctagataccatcaaga
pAOX1 genotyping	gactggttccaattgacaagc	
pPICZ B genotyping		gcaaatggcattctgacatcc
sfGFP genotyping		gttcctgcacatagccttcc
ManS genotyping	gagcaggagtcaggctcatc	tattgccatcttcctcaccag
Venus genotyping		gcatcaccttcaccttcacc
mRuby2 genotyping	gatacgaagatgggggtgtc	caaatgacctccaccatcaa
ScGOS1 genotyping	attctaacccggcaatctcc	
OCH1 genotyping	cgttttgtacggaccctcac	cgtccagtccatcacatcac
pGAP genotyping	tggtttctcctgacccaaag	
pFDH1 genotyping	ttcagtgtgctgacctacacg	
pDAS1 genotyping	gcctctaggcaaattctgttc	
pCAT1 genotyping	atgattttggcctgatgagc	
eGFP genotyping		aagtcgtgctgcttcatgtg
ScCYC1tt genotyping		cttttcggttagagcggatg
RPP1Btt genotyping		ggacaagccgtcactttcac
THD3tt genotyping		ttcaagccaatgctcaaatg
pAGT572_Nemo genotyping	ggggtaattaatcagcgaagc	gacaactagggccattgcag

Supporting Information

Supplemental References

(1) Robert, M.; Waldhauer, J.; Stritt, F.; Yang, B.; Pauly, M.; Voiniciuc, C. Modular Biosynthesis of Plant Hemicellulose and Its Impact on Yeast Cells. *Biotechnol. Biofuels* **2021**, *14* (1), 140. https://doi.org/10.1186/s13068-021-01985-z.

(2) Prielhofer, R.; Barrero, J. J.; Steuer, S.; Gassler, T.; Zahrl, R.; Baumann, K.; Sauer, M.; Mattanovich, D.; Gasser, B.; Marx, H. GoldenPiCS: A Golden Gate-Derived Modular Cloning System for Applied Synthetic Biology in the Yeast Pichia Pastoris. *BMC Syst. Biol.* **2017**, *11* (1), 123. https://doi.org/10.1186/s12918-017-0492-3.

(3) Püllmann, P.; Knorrscheidt, A.; Münch, J.; Palme, P. R.; Hoehenwarter, W.; Marillonnet, S.; Alcalde, M.; Westermann, B.; Weissenborn, M. J. A Modular Two Yeast Species Secretion System for the Production and Preparative Application of Unspecific Peroxygenases. *Commun. Biol.* **2021**, *4* (1), 1–20. https://doi.org/10.1038/s42003-021-02076-3.

(4) Jacobs, P. P.; Geysens, S.; Vervecken, W.; Contreras, R.; Callewaert, N. Engineering Complex-Type N-Glycosylation in Pichia Pastoris Using GlycoSwitch Technology. *Nat. Protoc.* **2009**, *4* (1), 58–70. https://doi.org/10.1038/nprot.2008.213.

(5) Voiniciuc, C.; Dama, M.; Gawenda, N.; Stritt, F.; Pauly, M. Mechanistic Insights from Plant Heteromannan Synthesis in Yeast. *Proc. Natl. Acad. Sci.* **2019**, *116* (2), 522–527. https://doi.org/10.1073/pnas.1814003116.

(6) Brachmann, C. B.; Davies, A.; Cost, G. J.; Caputo, E.; Li, J.; Hieter, P.; Boeke, J. D. Designer Deletion Strains Derived from Saccharomyces Cerevisiae S288C: A Useful Set of Strains and Plasmids for PCR-Mediated Gene Disruption and Other Applications. *Yeast* **1998**, *14* (2), 115–132. https://doi.org/10.1002/(SICI)1097-0061(19980130)14:2<115::AID-YEA204>3.0.CO;2-2.
